# Remote automated multi-generational growth and observation of an animal in low Earth orbit

**DOI:** 10.1098/rsif.2011.0716

**Published:** 2011-11-30

**Authors:** Elizabeth A. Oczypok, Timothy Etheridge, Jacob Freeman, Louis Stodieck, Robert Johnsen, David Baillie, Nathaniel J. Szewczyk

**Affiliations:** 1 Department of Biological Sciences, University of Pittsburgh, Pittsburgh, PA 15260, USA; 2 School of Graduate Entry Medicine and Health, University of Nottingham, Derby DE22 3DT, UK; 3 BioServe Space Technologies, University of Colorado, Boulder, CO 80309, USA; 4 Department of Molecular Biology and Biochemistry, Simon Fraser University, Burnaby, British Columbia, CanadaV5A 1S6

**Keywords:** *Caenorhabditis elegans*, spaceflight, astrobiology, interplanetary transfer

## Abstract

The ultimate survival of humanity is dependent upon colonization of other planetary bodies. Key challenges to such habitation are (patho)physiologic changes induced by known, and unknown, factors associated with long-duration and distance space exploration. However, we currently lack biological models for detecting and studying these changes. Here, we use a remote automated culture system to successfully grow an animal in low Earth orbit for six months. Our observations, over 12 generations, demonstrate that the multi-cellular soil worm *Caenorhabditis elegans* develops from egg to adulthood and produces progeny with identical timings in space as on the Earth. Additionally, these animals display normal rates of movement when fully fed, comparable declines in movement when starved, and appropriate growth arrest upon starvation and recovery upon re-feeding. These observations establish *C. elegans* as a biological model that can be used to detect changes in animal growth, development, reproduction and behaviour in response to environmental conditions during long-duration spaceflight. This experimental system is ready to be incorporated on future, unmanned interplanetary missions and could be used to study cost-effectively the effects of such missions on these biological processes and the efficacy of new life support systems and radiation shielding technologies.

## 1. Introduction

Colonization of other planets is deemed realistic [1]. This would mitigate against Earth's periodic global extinction events [2] and the next ice age; our nearest relative, Neanderthal, went extinct in the last ice age. Promisingly, the space-faring nations are planning long-duration expeditions beyond low Earth orbit (LEO) [3]. The economic cost is vast, estimated at several tens of billions of Euros [4], as technology must be developed to permit human presence on these missions (e.g. advanced life support and integrated sensing systems and superior radiation shielding). Accordingly, we currently know virtually nothing of the (patho)physiologic adaptations associated with habitation beyond LEO and, therefore, little of the long-term prospects for other worldly habitation.

Short-term studies within LEO demonstrate that (patho)physiologic changes occur when living in space [3,5]; radiation exposure and musculoskeletal deterioration are suggested as key obstacles to successful habitation beyond LEO. Animal models are recognized as cost-effective solutions to some problems intrinsic with studying humans (e.g. high cost, low throughput, slow discovery rate and endangerment of human health). The soil nematode *Caenorhabditis elegans* has been used on the Earth to help understand human biology [6]; its use for the space life sciences has also been demonstrated [7–9]. Briefly, *C. elegans*: has an evolved neuromuscular system; moves in three dimensions in soil or liquid; senses and responds behaviourally to its environment (detecting and moving towards or away from chemicals, heat, oxygen and ultraviolet radiation [10,11]) and is an accepted model for assaying environmental toxins [12]. Past spaceflight studies established that *C. elegans* and astronauts show similar alterations in muscle protein synthesis, particularly decreased synthesis of the contractile protein myosin and the transcription factor that controls myosin synthesis [13], that both appear to show alterations in insulin signalling [14], and that *C. elegans* can be used to detect in-flight radiation exposure [15–17]. While a worm is not a man, many spaceflight-induced molecular changes occur in both. Given the high cost of manned missions, *C. elegans* could be a cost-effective model for detecting, understanding, and mitigating some of the biological consequences of long-duration exploratory missions.

Here, we report the use of a remotely operated, automated culture system for *C. elegans* in LEO and demonstrate that it can be used to observe biological measures of animal health such as development, reproduction, behaviour and growth arrest as well as recovery during a long-duration spaceflight. This system could, in the future, be used on interplanetary missions.

## 2. Methods and results

### 2.1. Automated culturing and experimentation

**Figure 1.**
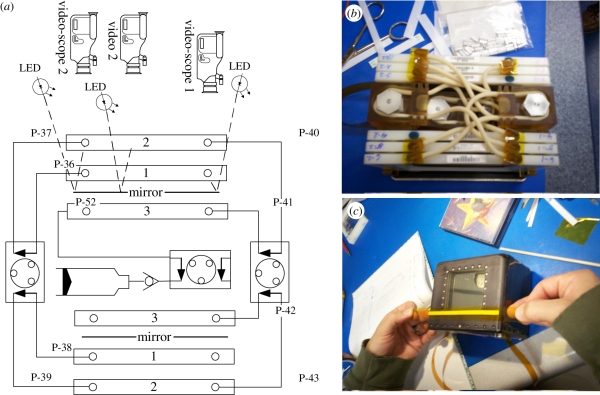
Automated culturing system for *C. elegans*. (*a*) Diagram of flow system for Opticells joined via peristaltic pumps and optical video system for observing animals. (*b*) Photograph of six joined Opticells prior to flight. (*c*) Photograph of culture system within housing prior to flight. (Online version in colour.)

Prior to flight, we developed an automated culturing system. We combined liquid *C. elegans* Maintenance Medium (CeMM), which supported normal growth and development of two generations of *C. elegans* on-board the International Space Station (ISS) [9], with the OptiCell (BioCrystal, Ltd.), which displayed no undesirable issues with spaceflight hardware [18]. OptiCells were linked together by infusion pump tubing and payload program controllable peristaltic pumps (figure 1); automated pump activation can be overridden by remote uplink commands. The linked OptiCells were housed within annodized aluminium fitted with a Plexiglas window. Visualization of animals through the window used LED illumination and board mounted miniature cameras (figure 1); Infinistix lenses (18 mm working distance, 2× primary magnification) magnified the field of view to approximately 3 × 4 mm. We confirmed no problems with remote operation of the pumps or cameras, or animal growth over one month on the Earth.

For spaceflight and ground controls, we used the Commercial Generic Bioprocessing Apparatus (CGBA) [19] to provide data downlink and temperature control (22 ± 1°C). We relied upon the ISS for oxygen and power in-flight and the University of Colorado for both on the Earth. Temperature, oxygen and relative humidity were monitored twice daily and remained within established parameters throughout six months in-flight and on the Earth.

### 2.2. Normal development and behaviour over 12 generations on-board the ISS

**Figure 2.**
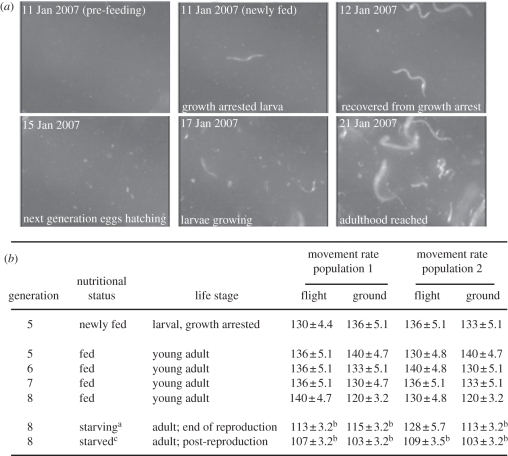
Normal development and behaviour of *C. elegans* in-flight. (*a*) Series of images, from video, display an Opticell receiving a growth-arrested larva (generation 5) from an initial Opticell, loaded with mixed stage animals pre-flight. After introduction to fresh food, the larvae escape growth arrest, develop to adulthood and lay eggs which also develop to adulthood (generation 6). (*b*) Videos were analysed for animal movement frequency (number of left-to-right head movements per minute). In generations 5–8, normal movement was observed for animals, recovered from growth arrest, in two independent populations. Movement declines upon starvation were also comparable to the Earth-based controls. Data displayed are mean ± s.e.m. (*n* = 100). ^a^Growing and growth-arrested ninth generation animals were observed. ^b^*p* < 0.001, two way repeated measures ANOVA (Graphpad Prism 5). ^c^No growing ninth or 10th generation animals were observed.

Daily observation of animals (figure 2*a*) confirmed the past inference that *C. elegans* develop normally in space [9,20,21]. Identical timings for egg to egg-laying adult were observed for the ground control and in-flight populations (figure 2*a*, 6.5 days at 22 ± 1°C which is consistent with past growth rates in CeMM on the Earth [18,22]). Lengths of animals measured in National Institutes of Health ImageJ software (*n* = 40 per day per condition, and additional measurements by grade and high-school students from the USA, Canada and Malaysia) confirm these developmental stages (such measurements accurately stage animals [22]).

*Caenorhabditis elegans* uses transforming growth factor-beta and insulin signalling to sense and respond to adverse environmental conditions such as lack of food and elevated temperature. These signals control entry into a developmentally arrested, stress-resistant, enduring state [23]. Because past gene expression studies suggested alteration in genes controlled by these signalling systems in spaceflown *C. elegans*, *Drosophilia* and men [14,24], we were concerned that cultivation of *C. elegans* beyond one to two generations might result in developmental arrest as the result of continued sensing of an adverse environment. However, in fed populations, we noted no changes in population distribution or developmental timing over 12 generations of growth in-flight (the first three months). Furthermore, worms were able to sense and respond appropriately to the presence of food in-flight as starved animals developmentally arrested and then recovered when introduced to fresh CeMM (figure 2).

*Caenorhabditis elegans* and man both display depressed synthesis of myosin, and other muscle gene products, in response to spaceflight and impaired mobility upon return to the Earth [13]. In-flight movement, when fed, was identical to that on the Earth (figure 2*b*), suggesting that decreases in muscular synthetic capacity are adaptive rather than pathological in-flight. These data suggest, but do not prove, that the same may be true for changes in human cardiac, skeletal and vascular muscles [3,5]. As we detected no movement decline over 12 generations, these results also suggest that the past concerns, that muscular decline may never plateau [3,5], may be false.

We also found decreased movement as food was depleted (figure 2*b*). This observation further suggests *C. elegans* remain able to respond appropriately to lack of food in space. Importantly, the movement decline and growth arrest also demonstrate that our system can be used to detect both normal and abnormal growth, development, reproduction and behaviour during spaceflight.

While we were able to recover viable populations after six months, delays with the Space Shuttle program precluded the observation of the full 24 generation experiment.

## 3. Discussion

We developed a compact automated *C. elegans* culturing system using off-the-shelf hardware. By combining this system with the established CGBA, we were able to remotely culture and observe *C. elegans* throughout 12 generations on-board the ISS. Consistent with past experiments in LEO, *C. elegans* display normal developmental timings when fed, and appropriate alterations when starved and re-fed. Accordingly, we were able to make the first observations of *C. elegans* behaviour in LEO. Over 12 generations, animals displayed normal movement rates when fed, and appropriate alterations when starved and re-fed. This demonstrates that it is possible for a multi-cellular animal to live long term in LEO (e.g. more than 10 generations) and that it can be studied, remotely, while in LEO. As *C. elegans* is an accepted model for assessing environmental toxins [12], including in-flight radiation [15–17], and as behavioural alterations continue to be an earlier indicator of toxic exposure than death [25], we suggest that *C. elegans* and the culturing system presented here are currently robust enough to consider incorporating biological specimens into future long-distance interplanetary missions. Given the high cost of manned space missions and high failure rate for Mars missions, we suggest that small organisms such as *C. elegans*, despite not being humans, be used as a cost-effective model for detecting, and potentially studying, some of the biological effects of long-duration and distance spaceflight. Our system provides a valuable test bed for life support system performance on missions where the risk of component failure is unacceptably high for manned missions.
